# Supplementary material for: Statistical methods for cis‐Mendelian randomization with two‐sample summary‐level data
Source: Genet Epidemiol. 2022 Oct 23;47(1):3–25. doi: 10.1002/gepi.22506 (PMC7614127; doi:10.1002/gepi.22506)
Supplement: Supplementary file 1 — Supplementary Information [file GEPI-47-3-s001.pdf]

Statistical Methods for cis-Mendelian Randomization with Two-sample Summary-level Data

Apostolos Gkatzionis[1,2*], Stephen Burgess[1,3], Paul J Newcombe[1]

[1]MRC Biostatistics Unit, University of Cambridge, UK.
[2]MRC Integrative Epidemiology Unit, University of Bristol, UK.
[3]Department of Public Health and Primary Care, School of Clinical Medicine, University of Cambridge, UK.

This file contains supplementary material for the paper "Statistical Methods for cis-Mendelian Randomization", currently submitted to Genetic Epidemiology.

Proportion of Variation Explained by Genetic Instruments

To fix the proportion v_G of variation in the risk factor explained by the genetic variants, we adjusted the residual variance σ_0^2 in our simulations accordingly. We first specified a value for v_G and generated SNP-risk factor associations β_{Xj} as described in the simulation design section of the manuscript. The value of σ_0^2 was then set equal to

$$\sigma_0^2 = \frac{1 - v_G}{2v_G} \frac{1}{N_{ref}} \beta_X^T G_{ref}^T G_{ref} \beta_X$$

where G_{ref} is the reference genetic matrix. This can be justified by recalling that in our simulation design,

$$X = \sum_{j=1}^{P} \beta_{Xj} G_j + \alpha_X U + \epsilon_X$$

with $U, \epsilon_X \sim N(0, \sigma_0^2)$. Taking variances, we have that $\text{Var}(X) = \text{Var}(G\beta_X) + 2\sigma_0^2$ and therefore,

$$\begin{aligned} v_G &= \frac{\text{Var}(G\beta_X)}{\text{Var}(X)} = \frac{\text{Var}(G\beta_X)}{\text{Var}(G\beta_X) + 2\sigma_0^2} \\ &= \frac{\frac{1}{N_{ref}} \beta_X^T G^T G \beta_X}{\frac{1}{N_{ref}} \beta_X^T G^T G \beta_X + 2\sigma_0^2} \end{aligned}$$

*Corresponding author. Email: apostolos.gkatzionis@bristol.ac.uk

which yields the previous expression for σ_0^2. A similar formula was derived in the appendix of Yang et al. (2012).

Additional Simulations - Large G-X Sample Size

To augment the analysis presented in the paper, we compared the various cis-MR methods in a range of additional simulations. This section contains simulation results for a simulation using a larger sample size of $N_1 = 100000$ to compute summary-level risk factor-outcome associations. This scenario may represent an applied analysis using a downstream biomarker as a proxy for protein expression, as this would allow researchers to obtain summary-level data from existing large-scale GWAS studies whose sample size often ranges in the hundreds of thousands. This was the case for the two real-data applications presented in the main part of our manuscript.

We conducted simulations both for the SHBG and for the HMGCR region, with six causal variants per region, as in our main simulations. For brevity, we only considered the "strong instruments" scenario, where $v_G = 3\%$ for the SHBG region and $v_G = 2\%$ for the HMGCR region. Otherwise, the simulations reported here were set up in the same way as those reported in the main part of our paper. Results are reported in Table 1.

A notable difference in the results compared to our main simulations was that a larger sample size resulted in stronger instruments. A tenfold increase in the sample size resulted in a similar, tenfold increase in the values of the F statistics. For a regression using only the six causal variants, the average F statistic was 514 for the SHBG and 340 for the HMGCR region. As a result, the various cis-MR methods were less affected by weak instruments bias and performed quite well. The performance of the stepwise pruning method was was much more consistent for different values of ρ than in the corresponding simulation with $N_1 = 10000$. PCA and JAM were both unaffected by weak instrument bias for $\theta = 0.1$. Confidence intervals constructed using these methods practically attained nominal coverage, and so did the confidence intervals based on F-LIML and CLR. A small reduction in power for single-SNP analysis and LD-pruning with low correlation thresholds were the only real difference between the various methods. This was the case both for the SHBG and for the HMGCR region.

These results imply that weak instrument bias is less likely to affect analyses based on large $G-X$ sample sizes, and stepwise pruning does not underperform other methods in such analyses.

Additional Simulations - Fewer Causal Variants

In our second set of additional simulations, we modified the number of causal variants that were present in each region. Focusing on the SHBG region, we considered two additional scenarios.

First, we assumed the existence of only a single causal variant in the region, placing the causal signal at the variant that had the smallest univariate p-value in our real-data application (rs1799941). That variant was assumed to have an effect of $\beta_{Xj} = 0.38$ on the risk factor, the same as that observed in our real SHBG-testosterone dataset.

In the second scenario, we generated three independent genetic effects on the risk factor. The effects were placed at genetic variants suggested as independently causal by Jin et al. (2012), who analysed genetic associations of variants in the SHBG region with serum testosterone levels. Genetic effects for the causal variants were drawn randomly according to $\beta_{Xj} \sim |N(0, 0.2)| + 0.1$ for the

Table 1: Performance of cis-MR methods in simulations for various values of the causal effect parameter θ, using genetic variants from two gene regions (SHBG and HMGCR), "strong" genetic instruments (corresponding F statistics ¿ 10) and a large $G-X$ sample size ($N_1 = 100000$).

Method		$\theta=0$			$\theta=0.05$				$\theta=0.1$		
		$\hat{\theta}$	$se(\hat{\theta})$	Type I	$\hat{\theta}$	$se(\hat{\theta})$	Cov	Power	$\hat{\theta}$	$se(\hat{\theta})$	Cov
SHBG Region											
Top SNP	—	-0.001	0.019	0.063	0.050	0.020	0.951	0.710	0.101	0.020	0.952
Pruning	$\rho=0.1$	-0.001	0.016	0.054	0.050	0.016	0.943	0.868	0.100	0.016	0.954
	$\rho=0.3$	-0.001	0.014	0.039	0.050	0.014	0.946	0.940	0.099	0.015	0.958
	$\rho=0.5$	-0.001	0.014	0.041	0.050	0.014	0.946	0.949	0.099	0.014	0.956
	$\rho=0.7$	-0.001	0.013	0.033	0.049	0.014	0.947	0.944	0.098	0.014	0.952
	$\rho=0.9$	-0.001	0.013	0.036	0.048	0.013	0.949	0.941	0.095	0.014	0.941
PCA	$k=0.99$	0.000	0.015	0.038	0.051	0.015	0.950	0.909	0.100	0.016	0.957
	$k=0.999$	0.000	0.014	0.040	0.051	0.015	0.944	0.939	0.100	0.015	0.959
JAM	$\rho=0.6$	-0.001	0.014	0.037	0.050	0.014	0.947	0.951	0.100	0.014	0.959
	$\rho=0.8$	-0.001	0.013	0.038	0.051	0.014	0.949	0.948	0.100	0.014	0.953
	$\rho=0.9$	0.000	0.013	0.040	0.051	0.014	0.947	0.952	0.100	0.014	0.960
	$\rho=0.95$	0.000	0.013	0.038	0.051	0.014	0.952	0.951	0.100	0.014	0.960
F-LIML	—	-0.001	0.014	0.046	0.051	0.015	0.952	0.919	0.100	0.015	0.961
CLR	—	—	—	0.044	—	—	0.949	0.919	—	—	0.958
HMGCR Region											
Top SNP	—	0.000	0.019	0.055	0.050	0.019	0.950	0.738	0.100	0.020	0.951
Pruning	$\rho=0.1$	0.000	0.018	0.048	0.050	0.018	0.956	0.769	0.100	0.019	0.956
	$\rho=0.3$	0.000	0.017	0.051	0.049	0.018	0.947	0.795	0.099	0.018	0.951
	$\rho=0.5$	0.000	0.017	0.048	0.050	0.017	0.950	0.818	0.099	0.018	0.941
	$\rho=0.7$	0.000	0.016	0.048	0.049	0.017	0.947	0.836	0.098	0.017	0.942
	$\rho=0.9$	0.000	0.016	0.048	0.048	0.017	0.956	0.821	0.096	0.017	0.938
PCA	$k=0.99$	0.000	0.017	0.047	0.050	0.017	0.956	0.815	0.100	0.018	0.947
	$k=0.999$	0.000	0.017	0.049	0.050	0.017	0.956	0.819	0.100	0.018	0.948
JAM	$\rho=0.6$	0.000	0.017	0.049	0.050	0.017	0.952	0.816	0.099	0.018	0.937
	$\rho=0.8$	0.000	0.017	0.048	0.050	0.017	0.955	0.827	0.100	0.017	0.942
	$\rho=0.9$	0.000	0.017	0.045	0.050	0.017	0.957	0.829	0.099	0.017	0.938
	$\rho=0.95$	0.000	0.017	0.049	0.050	0.017	0.951	0.825	0.099	0.018	0.942
F-LIML	—	0.000	0.017	0.049	0.051	0.017	0.952	0.825	0.100	0.018	0.949
CLR	—	—	—	0.049	—	—	0.947	0.823	—	—	0.947

Table 2: Performance of cis-MR methods in simulations for various values of the causal effect parameter θ, using genetic variants from the SHBG gene region, "strong" genetic instruments (corresponding F statistics ¿ 10) and either 1 or 3 causal variants.

		$\theta = 0$			$\theta = 0.05$				$\theta = 0.1$		
Method		$\hat{\theta}$	$se(\hat{\theta})$	Type I	$\hat{\theta}$	$se(\hat{\theta})$	Cov	Power	$\hat{\theta}$	$se(\hat{\theta})$	Cov
One Causal Variant											
Top SNP	—	0.001	0.013	0.046	0.051	0.014	0.952	0.966	0.100	0.014	0.937
Pruning	$\rho = 0.1$	0.001	0.013	0.046	0.051	0.014	0.952	0.966	0.100	0.014	0.937
	$\rho = 0.3$	0.001	0.013	0.047	0.050	0.014	0.953	0.961	0.099	0.014	0.932
	$\rho = 0.5$	0.001	0.013	0.045	0.049	0.014	0.949	0.956	0.096	0.014	0.920
	$\rho = 0.7$	0.001	0.013	0.047	0.047	0.013	0.947	0.959	0.093	0.014	0.899
	$\rho = 0.9$	0.001	0.012	0.050	0.043	0.013	0.918	0.934	0.086	0.013	0.767
PCA	$k = 0.99$	0.000	0.015	0.053	0.050	0.016	0.946	0.882	0.099	0.016	0.924
	$k = 0.999$	0.001	0.014	0.051	0.048	0.014	0.952	0.922	0.096	0.015	0.919
JAM	$\rho = 0.6$	0.001	0.013	0.046	0.051	0.014	0.952	0.965	0.100	0.014	0.938
	$\rho = 0.8$	0.001	0.013	0.046	0.051	0.014	0.952	0.966	0.100	0.014	0.938
	$\rho = 0.9$	0.001	0.013	0.046	0.051	0.014	0.953	0.966	0.100	0.014	0.938
	$\rho = 0.95$	0.001	0.013	0.046	0.051	0.014	0.953	0.966	0.100	0.014	0.938
F-LIML	—	0.001	0.014	0.061	0.051	0.015	0.944	0.936	0.100	0.016	0.942
CLR	—	—	—	0.047	—	—	0.951	0.928	—	—	0.955
Three Causal Variants											
Top SNP	—	0.000	0.016	0.051	0.050	0.016	0.956	0.852	0.098	0.017	0.934
Pruning	$\rho = 0.1$	0.000	0.015	0.047	0.049	0.016	0.953	0.868	0.097	0.016	0.928
	$\rho = 0.3$	0.000	0.014	0.046	0.049	0.014	0.942	0.919	0.096	0.015	0.930
	$\rho = 0.5$	0.000	0.013	0.049	0.047	0.014	0.945	0.928	0.095	0.014	0.915
	$\rho = 0.7$	0.000	0.013	0.046	0.046	0.013	0.931	0.934	0.091	0.014	0.867
	$\rho = 0.9$	0.000	0.012	0.042	0.041	0.013	0.891	0.904	0.083	0.013	0.724
PCA	$k = 0.99$	0.000	0.015	0.051	0.049	0.015	0.941	0.884	0.096	0.016	0.932
	$k = 0.999$	0.000	0.014	0.047	0.047	0.014	0.944	0.905	0.095	0.015	0.925
JAM	$\rho = 0.6$	0.000	0.014	0.046	0.049	0.014	0.945	0.939	0.097	0.015	0.930
	$\rho = 0.8$	0.000	0.014	0.046	0.049	0.014	0.946	0.938	0.097	0.014	0.929
	$\rho = 0.9$	0.000	0.014	0.046	0.049	0.014	0.942	0.943	0.097	0.014	0.923
	$\rho = 0.95$	0.000	0.013	0.044	0.049	0.014	0.946	0.948	0.097	0.014	0.929
F-LIML	—	0.000	0.014	0.060	0.050	0.014	0.932	0.933	0.100	0.016	0.949
CLR	—	—	—	0.049	—	—	0.940	0.919	—	—	0.959

risk-increasing allele, same as what we did for the six-causal-variants simulation in the main part of our paper.

Otherwise, the simulations were set up as previously described. We used the "strong instruments" scenario with $v_G = 3\%$ and a $G - X$ sample size of $N_1 = 10000$. Simulation results are reported in Table 2.

The results closely resembled the ones for our original simulation scenario with "strong instruments" and six causal variants. All methods were quite accurate for $\theta = 0$. When $\theta \neq 0$, stepwise pruning was subject to weak instrument bias to some extent, especially for large correlation thresholds. The performance of the method depended on the correlation threshold used. PCA and JAM were more consistent in terms of their tuning parameters and were generally quite accurate, while factor-based methods were even more accurate, with a small inflation of Type I error rates under the null for F-LIML.

Most methods performed slightly better with one than with three causal variants, but the differ-

ences in their performance were small and it seems that the number of causal signals in the region should have little impact on the choice of which cis-MR method to use. An exception relates to the use of the top-SNP approach, which was expectedly quite accurate when only a single causal variant existed in the region. With three causal variants, top-SNP analysis was subject to the same issues as in our original simulations (namely larger standard errors and lower power than other methods), but to a lesser degree.

Additional Simulations - Small Reference Dataset

In our third set of additional simulations, we wanted to assess the impact of the reference dataset on the various MR methods. In particular, we assessed whether the use of a small reference dataset, which would imply inaccurate genetic correlation estimates, affects some cis-MR methods worse than others. We therefore repeated the simulations from the main part of our paper, but bootstrapped the rows of the UK Biobank matrix and only selected $N_{ref} = 1000$ individuals from which to compute genetic correlations, instead of using the entire UK Biobank dataset of $N_{bb} = 367643$ individuals. In practice, using small datasets as reference data is not uncommon: for example, the 1000 genomes dataset is commonly used for this purpose. The use of a small reference dataset does not systematically bias the estimated genetic covariance matrix, as would be the case with population stratification.

We used both genetic regions and the “strong instruments” scenario. Again, the simulation design was identical to those reported in the main part of the paper, except using a smaller reference dataset. Results are reported in Table 3.

The JAM algorithm proved to be more sensitive to the reference dataset. The algorithm adjusts marginal SNP-trait associations using the reference genetic correlations. By using an inaccurate correlation pattern, the algorithm’s adjustment was adversely affected and this resulted in selecting more genetic variants than in runs with a larger reference dataset. This was especially the case for large values of the correlation threshold, in which case the pre-pruning step only discards a small number of variants before running JAM. For example, for the SHBG region with $\theta = 0$ and $\rho = 0.95$, the algorithm had a posterior model size of 9.27 compared to 3.75 when the entire UK Biobank was used as a reference dataset. This meant that the algorithm was more susceptible to weak instrument bias, made JAM causal effect estimates slightly more variable for larger ρ values and increased Type I error rates. For smaller values of the correlation threshold the algorithm’s performance was affected less.

The performance of LD-pruning also attenuated for large values of its correlation threshold, but this attenuation was in line with what we observed in our original simulations, using a large reference dataset. Likewise, principal components analysis and factor-based methods seemed fairly robust to using a smaller reference dataset.

Table 3: Performance of cis-MR methods in simulations for various values of the causal effect parameter θ, using genetic variants from two gene regions (SHBG and HMGCR), "strong" genetic instruments (corresponding F statistics ¿ 10) and a smaller reference sample ($N_{ref} = 1000$).

Method		$\theta = 0$			$\theta = 0.05$				$\theta = 0.1$		
		$\hat{\theta}$	$se(\hat{\theta})$	Type I	$\hat{\theta}$	$se(\hat{\theta})$	Cov	Power	$\hat{\theta}$	$se(\hat{\theta})$	Cov
SHBG Region											
Top SNP	——	-0.001	0.019	0.039	0.048	0.019	0.941	0.676	0.095	0.020	0.917
Pruning	$\rho = 0.1$	-0.001	0.016	0.046	0.048	0.016	0.925	0.799	0.095	0.017	0.892
	$\rho = 0.3$	0.000	0.014	0.045	0.048	0.014	0.942	0.900	0.095	0.015	0.901
	$\rho = 0.5$	0.000	0.013	0.049	0.047	0.014	0.933	0.914	0.093	0.014	0.892
	$\rho = 0.7$	0.000	0.013	0.048	0.046	0.013	0.917	0.916	0.090	0.014	0.863
	$\rho = 0.9$	0.000	0.012	0.057	0.043	0.012	0.871	0.900	0.084	0.013	0.750
PCA	$k = 0.99$	0.000	0.015	0.041	0.049	0.015	0.939	0.883	0.097	0.015	0.922
	$k = 0.999$	0.000	0.014	0.052	0.048	0.014	0.925	0.904	0.094	0.015	0.919
JAM	$\rho = 0.6$	0.000	0.014	0.038	0.047	0.014	0.937	0.896	0.093	0.015	0.906
	$\rho = 0.8$	0.000	0.014	0.039	0.047	0.014	0.922	0.896	0.092	0.014	0.887
	$\rho = 0.9$	0.000	0.014	0.063	0.047	0.014	0.910	0.895	0.092	0.014	0.867
	$\rho = 0.95$	0.000	0.014	0.093	0.047	0.014	0.888	0.885	0.091	0.014	0.843
F-LIML	——	0.000	0.014	0.052	0.050	0.014	0.921	0.907	0.099	0.016	0.929
CLR	——	——	——	0.040	——	——	0.929	0.895	——	——	0.933
HMGCR Region											
Top SNP	——	-0.001	0.018	0.047	0.048	0.018	0.951	0.753	0.099	0.019	0.921
Pruning	$\rho = 0.1$	-0.001	0.018	0.048	0.048	0.018	0.951	0.757	0.099	0.019	0.919
	$\rho = 0.3$	-0.001	0.017	0.056	0.047	0.017	0.951	0.770	0.097	0.018	0.928
	$\rho = 0.5$	-0.001	0.016	0.052	0.047	0.017	0.947	0.807	0.096	0.017	0.920
	$\rho = 0.7$	-0.001	0.016	0.052	0.046	0.016	0.943	0.818	0.093	0.017	0.893
	$\rho = 0.9$	0.000	0.014	0.113	0.042	0.015	0.836	0.785	0.084	0.015	0.735
PCA	$k = 0.99$	-0.001	0.017	0.047	0.048	0.017	0.950	0.816	0.098	0.018	0.921
	$k = 0.999$	0.000	0.016	0.051	0.047	0.017	0.949	0.814	0.097	0.017	0.922
JAM	$\rho = 0.6$	-0.001	0.017	0.047	0.047	0.018	0.949	0.782	0.096	0.018	0.933
	$\rho = 0.8$	-0.001	0.017	0.062	0.046	0.017	0.934	0.794	0.094	0.018	0.910
	$\rho = 0.9$	0.000	0.017	0.093	0.045	0.017	0.895	0.790	0.093	0.018	0.859
	$\rho = 0.95$	-0.001	0.017	0.150	0.044	0.017	0.824	0.759	0.092	0.018	0.791
F-LIML	——	-0.001	0.016	0.052	0.049	0.017	0.941	0.825	0.101	0.019	0.945
CLR	——	——	——	0.049	——	——	0.945	0.810	——	——	0.942

References

Jin, G., J. Sun, S.-T. Kim, J. Feng, Z. Wang, S. Tao, Z. Chen, L. Purcell, S. Smith, W. B. Isaacs, R. S. Rittmaster, S. L. Zheng, L. D. Condreay, and J. Xu (2012). Genome-wide association study identifies a new locus JMJD1C at 10q21 that may influence serum androgen levels in men. *Human Molecular Genetics 21*(23), 5222–5228.

Yang, J., T. Ferreira, A. P. Morris, S. E. Medland, G. I. of ANthropometric Traits (GIANT) Consortium, D. G. Replication, M. analysis (DIAGRAM) Consortium, P. A. F. Madden, A. C. Heath, N. G. Martin, G. W. Montgomery, M. N. Weedon, R. J. Loos, T. M. Frayling, M. I. McCarthy, J. N. Hirschhorn, M. E. Goddard, and P. M. Visscher (2012). Conditional and joint multiple-SNP analysis of GWAS summary statistics identifies additional variants influencing complex traits. *Nature Genetics 44*(4), 369–375.
